# Atrioventricular Block Treatment: Pacing Site, AV Synchrony, or Both?

**DOI:** 10.3390/jcm14030980

**Published:** 2025-02-04

**Authors:** Mauro Biffi, Annalisa Bagatin, Alberto Spadotto, Mirco Lazzeri, Alessandro Carecci, Lorenzo Bartoli, Cristian Martignani, Andrea Angeletti, Igor Diemberger, Giulia Massaro, Michele Bertelli, Matteo Ziacchi

**Affiliations:** 1 Institute of Cardiology, IRCCS Azienda Ospedaliero-Universitaria di Bologna, Via Massarenti 9, 40138 Bologna, Italy; 2 Department of Medical and Surgical Sciences, Institute of Cardiology, University of Bologna, Via Massarenti 9, 40138 Bologna, Italy

**Keywords:** leadless, Micra AV, atrioventricular synchrony, His bundle pacing, right ventricular pacing

## Abstract

**Background/Objectives**: Right ventricular pacing (RVP), leadless pacing (LL), and conduction system pacing (CSP) are treatment options for atrioventricular block (AVB), each with distinct characteristics. However, the long-term outcomes of these pacing strategies remain insufficiently compared. This study evaluates clinical and echocardiographic outcomes of patients with AVB treated with dual chamber RVP, His bundle pacing (HBP), or LL. **Methods**: This single-center observational registry study included 22 consecutive patients receiving LL with atrioventricular resynchronization functionality (October 2020 to October 2022), matched with 66 control patients receiving either RVP (33 patients) or HBP (33 patients) using propensity score matching (2:3:3 ratio). Primary and secondary endpoints included all-cause mortality, cardiovascular mortality, heart failure, and echocardiographic outcomes. Atrioventricular synchrony in the LL group was assessed. **Results**: At two years, all-cause mortality was significantly higher in the LL group compared to RVP (36.4% vs. 6.1%, *p* = 0.002) and HBP (36.4% vs. 12.1%, *p* = 0.03), but LL had a more severe clinical profile. Cardiovascular mortality and heart failure incidence showed no significant differences. Patients receiving RVP showed a significant decrease in left ventricular ejection fraction and an increase in ventricular volumes. In contrast, HBP patients exhibited favorable cardiac remodeling. Stratification based on atrial sensing showed that LL patients with >66% AV synchrony had a lower mortality (*p* = 0.02). **Conclusions**: CSP offers superior results compared to other pacing methods in terms of ventricular function owing to a physiological ventricular activation and maintenance of AV synchrony. However, LL may be a viable alternative for frail and high-risk patients, as the suboptimal AV synchrony is traded off with lesser ventricular dyssynchrony.

## 1. Introduction

Conventional right ventricular pacing (RVP), leadless pacing (LL), and conduction system pacing (CSP) are treatment options for atrioventricular block (AVB), each with distinct characteristics [1,2,3]. They differ in terms of the technical expertise required, effects on ventricular mechanical performance, and the type and severity of long-term complications.

The right ventricular apex has been the conventional site for pacemaker implantation since the early days of pacing. Chronic apical pacing may lead to both inter- and intra-ventricular dyssynchrony and is associated with an increased risk of heart failure (HF), atrial fibrillation (AF), and mortality, particularly when ventricular pacing exceeds 20% [4,5,6,7]. Right ventricular septal pacing has been proposed as a potential alternative; however, studies regarding its benefits remain inconclusive [8,9]. In the study by Kiehl et al. [10], a right ventricular pacing burden of ≥20% was significantly associated with the development of pacing-induced cardiomyopathy, irrespective of the pacing site (apical or septal).

His bundle pacing (HBP), by stimulating the His-Purkinje system, is the most “physiological” pacing modality, through maintaining the physiologic ventricular activation [11]. HBP has been shown to reduce the incidence of HF and AF compared to right ventricular pacing (RVP) [12,13,14,15]. However, the use of HBP has some limitations, such as over- and under-sensing and an increase in the capture threshold [16], which have hindered its widespread adoption. Particularly, the competing use of left bundle branch area pacing (LBBAP) has emerged as an alternative pacing strategy for the conduction system [17].

Leadless pacing (LL) is an alternative to transvenous pacemakers in specific subgroups of patients, such as those without superior access to the heart or those at high risk of infection or bleeding complications [1,18]. First-generation leadless pacemakers were limited to the VVI(R) mode due to their inability to sense atrial activation. This limitation was addressed with the introduction of the Micra AV, a ventricular leadless VDD pacemaker designed to maintain atrioventricular (AV) synchrony by tracking atrial mechanical activity [19]. More recently, leadless pacemakers designed for atrial placement have also been introduced, further expanding the potential applications of LL [20].

No randomized data are available to directly compare the outcomes of these three pacing strategies, and the existing evidence remains limited.

The aim of the study was to conduct a comparison with patients who received an RVP, HBP, or LL pacemaker at our center, focusing on clinical and echocardiographic outcomes.

## 2. Materials and Methods

### 2.1. Study Design and Patient Selection

This was a prospective study enrolling all consecutive patients with AVB who underwent the implantation of a leadless pacemaker with atrioventricular resynchronization functionality (Micra AV, Medtronic Inc., Minneapolis, MN, USA), from October 2020 to October 2022 at our center (Sant’Orsola-Malpighi Hospital, Bologna, Italy), as part of a single-center registry. Each Micra AV patient was matched with control patients treated with either dual-chamber RVP or HBP for AVB in a 2:3:3 ratio using propensity score matching. Propensity matching included age, sex, hypertension, diabetes, coronary artery disease, and NYHA class. Dialysis could not be a matching criterion, as it is a preferential characteristic for LL implantation in our center, according to our former experience [18]. These patients were implanted at our center during the same period. Each patient underwent LL, CSP, or RVP based on a clinical decision by our team according to our former experience with LL [18]. Beyond patients without superior access to the heart, in our center, LL was considered in frail patients with a high bleeding or infective risk, after a transvenous device extraction, and in those patients who were in need of only sporadic pacing (<20% of the time) [18].

The primary endpoint was all-cause mortality at a two-year follow-up, analyzed using survival curves. An additional regression analysis was conducted to evaluate whether, after propensity score adjustment, the baseline characteristics that differed between the groups impacted overall mortality.

Secondary endpoints included cardiovascular mortality and the incidence of HF (defined as hospitalization for HF or onset of typical HF signs and symptoms, leading to unplanned medical contact and therapeutic intervention). Additionally, periprocedural complications were evaluated, as well as the evolution of left ventricular ejection fraction and ventricular volumes as assessed by echocardiography. Lastly, additional analysis on all-cause mortality was elaborated by dividing patients into three categories based on the amount of time AV synchrony was obtained for each patient (0–33%; 34–66%; 67–100%). AV synchrony was defined as a 1:1 ratio between an intrinsic or a paced atrial and ventricular activation within a 300 ms interval, according to Steinwender et al. [19], based on electrocardiogram (EKG) recording in the clinic and repeated Holter (PC 6.03, General Electric, Boston, MA, USA) recordings at 1-month, 12-month, and 2-year follow-ups.

All LL patients had the lower rate programed at 35 bpm to avoid VVI pacing at the lower rate, and the maximum tracking rate at 140 bpm.

All patients were enrolled in an observational registry approved by our Institutional Review Boards, and the study adhered to the guidelines of the Declaration of Helsinki.

### 2.2. Data Collection and Clinical Follow-Up

The initial clinical evaluation included an EKG, echocardiogram, and chest X-ray. During the implantation procedure, sensing values, thresholds, and pacing impedances were recorded.

As per the clinical practice of our center, all patients underwent regular follow-ups.

Specifically, in-clinic pacemaker follow-ups were scheduled for 1 month and 12 months post-implantation, and subsequently every 12 months. During each visit, electrical parameters, device-recorded events, and any system modifications as dictated by clinical needs were recorded. A 24-h Holter was recorded at the end of in-clinic follow-ups to investigate the level of AV synchrony. Patients were given remote monitors as enabled by the implanted device. Arrhythmic events and their duration, along with follow-ups, were recorded either during in-clinic pacemaker follow-ups or via remote monitoring.

Echocardiograms were performed at baseline (prior to implantation) and at 12 months post-implantation to assess cardiac remodeling.

### 2.3. Statistical Analysis

Continuous data are presented as the mean ± standard deviation (SD) for normally distributed variables or as median ± interquartile range (IQR) for non-normally distributed data. Categorical variables are reported as absolute frequencies and relative percentages.

Continuous variables were then evaluated with the Mann–Whitney U test for non-parametric data, while the Kruskal–Wallis test was used when comparing more than two groups. Categorical variables, in turn, were compared with Chi-square tests of independence between groups.

Survival analysis was performed using Kaplan–Meier estimates, and survival distributions between groups were compared using the log-rank test. A proportional hazards assumption was tested using the Schoenfeld test. A Cox regression analysis was performed to evaluate independent predictors of all-cause mortality.

A *p*-value < 0.05 was considered statistically significant. Analyses were conducted using STATA (version 18.0, StataCorp LLC, College Station, TX, USA).

## 3. Results

### 3.1. Study Population

From October 2020 to October 2022, 22 patients with atrioventricular block were implanted with a Micra AV (Medtronic Inc., Minneapolis, MN, USA) LL. Six of the 22 patients (27%) who received a Micra AV had previously undergone pacemaker extraction due to CIED infection. In all the other patients, the pacemaker implantation was a first-time procedure.

The baseline characteristics are described in Table 1. The average age of the population at the time of implantation was 74.2 years, and 30.7% of the patients enrolled in this study were female. The three groups (LL, RVP, and HBP) exhibited similar clinical and echocardiographic characteristics, except for dialysis and chronic kidney disease (CKD, defined as a GFR < 60 mL/min or creatinine > 1.4 mg/dL), a history of treated valvular disease, and history of atrial fibrillation, which were slightly more prevalent in the LL group.

The choice of implantation type was determined by the patient’s profile and physician’s judgment. The primary reason for selecting a LL was as follows: 13 patients (59%) due to a high risk of infection (including 6 patients who had undergone prior device extraction for infection), 6 patients (27%) due to chronic kidney disease (CKD) requiring dialysis, 2 patients (9%) due to the absence of upper venous access, and 1 patient (5%) due to a high bleeding risk. All LL pacemakers are implanted at the septal location to minimize both the risk of pacing-induced cardiomyopathy and ventricular perforation/pericardial effusion.

### 3.2. Clinical Outcomes

**Figure 1:**
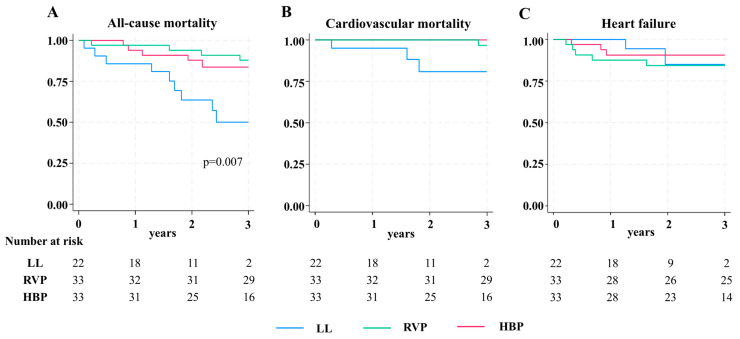
Kaplan–Meier event-free survival curves stratified by pacemaker type: RVP, HBP, and LL. (**A**) All-cause mortality, (**B**) Cardiovascular mortality, and (**C**) Heart failure. HPB = His bundle pacing, LL = leadless pacemaker, RVP = right ventricular pacing.

The primary endpoint of 2-year mortality differed significantly among the three groups (*p* = 0.007) (Figure 1A). Specifically, the RVP group exhibited lower overall mortality compared to the LL group (6.1% vs. 36.4%; HR 0.17; 95% CI 0.05–0.53, *p* = 0.002). Similarly, the HBP group demonstrated lower mortality compared to the LL group (12.1% vs. 36.4%; HR 0.32; 95% CI 0.12–0.88, *p* = 0.03). No significant difference in mortality was observed between the RVP and HBP groups. The Schoenfeld test confirmed the proportionality of hazard risk: *p* = 0.95.

In the univariate regression analysis of the characteristics that were statistically different at baseline among the three groups under investigation, factors such as CKD on dialysis and a history of atrial fibrillation were associated with higher overall mortality. However, in the multivariate analysis, only dialysis remained significantly associated (Table 2).

When analyzing cardiovascular mortality alone, no significant differences were observed among the three groups (Figure 1B). Similarly, no differences were found among the groups regarding the incidence of HF (Figure 1C).

When evaluating patients with LL based on the indications for implantation, those implanted due to dialysis appeared particularly vulnerable, exhibiting an all-cause mortality rate of 44% at two years. Notably, none of the patients who received an LL following the extraction of a transvenous pacemaker died during the follow-up period. However, due to the small sample size, meaningful comparisons within the groups are not possible.

### 3.3. Echocardiographic Outcomes

Baseline echocardiographic parameters presented no significant differences among the three populations. During the 12-month post-implantation echocardiography, the LL group had an unchanged LV volume and EF compared to the baseline (Table 3). On the other hand, HBP recipients had a statistically significant decrease in end-diastolic and end-systolic volume, while RVP recipients exhibited a decrease in left ventricular ejection fraction (LVEF) and an increase in both end-diastolic and end-systolic volume (Table 3). The proportion of patients with moderate or severe MR was also increased in RVP recipients (Table 3).

### 3.4. Peri-Procedural and Long-Term Complications

In the entire population, there were three (3.4%) peri-procedural complications, including two pneumothoraxes (2.3%), and one vascular access hematoma (1.1%) in a LL recipient; one patient required a chest tube, the other two complications were uneventful. During follow-up, there was only one (1.1%) lead dislodgement and two (2.3%) pacing threshold increases above 1.75 V (one in the RVP group and one in the HBP group). No statistically significant differences were found in the number and type of complications among the three groups in our study. Both periprocedural and long-term complications are summarized in Table 4.

### 3.5. Atrioventricular Synchrony

In the population of our study, AV synchrony was maintained in 100% of patients in the RVP and HBP groups, as the indication for implantation was an advanced atrioventricular block. In patients who received an LL, designed to detect the mechanical contraction of the right atrium rather than the electrical signal, the preservation of AV synchrony was not always achievable in an optimal manner despite repeated attempts at optimizing atrial activity detection as defined by Garweg et al. [21]. Atrioventricular synchrony was analyzed at three different follow-up times in LL recipients: t1 (1 month after implantation), t2 (12 months after implantation), and t3 (2 years after implantation). The percentage of AV synchrony in the LL population showed significant variability, ranging from values greater than 90% to nearly 0%. The average AV synchrony was 38.2% at t1, which slightly decreased to 30.2% at t2, and then improved at t3, reaching 37.9%.

**Figure 2:**
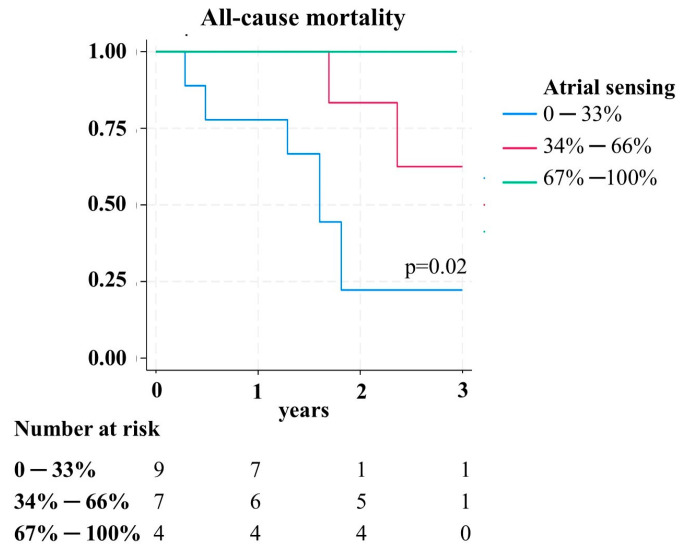
Kaplan–Meier event-free survival curves stratified by atrial sensing percentage in patients with an LL.

Stratifying the survival rate of LL patients based on the percentage of atrial sensing at t1, the group with more than 66% synchronous stimulation showed significantly lower mortality compared to patients with synchronous stimulation ≤ 33% (*p* = 0.02) (Figure 2).

The extent of stimulation differed significantly across the three groups (Table 5), with 6/22 (27%) LL recipients receiving < 20% RV pacing vs. none in the RVP and HBP groups.

## 4. Discussion

The treatment of patients with AVB has been a matter of debate in the past 30 years, focusing on its pattern (paroxysmal vs. permanent) and the individual patient’s profile: based on the extent of RV stimulation and the underlying LV systolic function the clinical outcome may be dramatically different. To date, the unsolved debate about the need to maintain AV synchrony and to achieve a physiological activation is challenged also by the quest to minimize intravascular hardware, as enabled by leadless pacemakers [22]. While the superiority of HBP with respect to customary RV pacing is being tested in randomized controlled trials, the comparison with LL pacing is not likely to occur in the next years.

In this single-center study, we observed that maintenance of AV synchrony and physiologic ventricular activation are both key determinants of cardiac function in an aged population, but in non-pacemaker dependent patients a suboptimal AV synchrony such as that enabled by VDD leadless pacers may be an acceptable trade-off. Indeed, the highest observed mortality of LL recipients was driven by non-cardiovascular events rather than by LV dysfunction.

The patient groups showed similar clinical characteristics due to propensity score matching and were evaluated over a homogeneous follow-up period, but a higher-risk profile in the LL group was not avoidable owing to the specific situations dictating intravascular hardware minimization, such as dialysis, advanced renal insufficiency, high infectious risk, and other comorbidities. On the other hand, LL recipients were also far less pacemaker-dependent than the RV and HBP groups (Table 5).

All three implantation techniques proved to be safe, with very low rates of periprocedural and late-onset complications. The duration of the follow-up was insufficient to demonstrate an advantage of LL over RVP and HBP in terms of late-onset complications, particularly lead-related issues, likely due to the short follow-up period.

### 4.1. Maintenance of Atrioventricular Synchrony

While transvenous systems, whether HBP or conventional RVP, use the atrial electrical signal to synchronize ventricular pacing, the leadless pacemaker detects the atrial mechanical activity corresponding to the fourth heart sound, using the pacemaker’s accelerometer sensor. Clearly, these two different methodologies can produce different results, especially in the presence of high atrial rates, which challenge the distinction between the third and fourth heart sounds [21]. Indeed, we observed that atrioventricular synchrony occurred 100% of the time for all patients treated with transvenous pacemakers, while in LL patients, AV synchrony, defined as QRS onset within 300 ms from sensed atrial activity, was observed only for about 40% of the time on average. Moreover, in the latter group, it was observed that those who maintained AV synchrony for more than 66% of the time had better prognoses when compared to those with poor AV synchrony: specifically, an inverse relationship between the percentage of AV synchrony and mortality was observed. This inference could certainly be debatable, as the reported study was not adequately powered to assess this outcome, thus, this observation must be considered exploratory; however, this result is consistent with cardiovascular physiology and with randomized clinical trials conducted over the past 25 years [23,24,25,26,27,28]. As highlighted by these studies, AV synchrony is an important factor in maintaining the cardiac index and left ventricular function [29], but it doesn’t represent the only determinant of cardiac performance, as ventricular activation impacts mechanical efficiency. Ventricular activation due to right ventricular myocardial pacing, rather than by the His-Purkinje system, leads to contraction dyssynchrony that adversely affects the systolic and diastolic performance of the left ventricle. In fact, in the previously mentioned studies, 13% of patients treated with traditional RVP developed left ventricular systolic dysfunction and/or heart failure during follow-ups as a consequence of the altered ventricular activation [10]. This aspect was confirmed in our population, where an increase in volume and a reduction in LVEF occurred in the RVP-treated group of patients. On the other hand, the results were different when compared to patients with HBP, whose physiological ventricular activation resulted in an improved volume and cardiac function. Patients with leadless pacing fell somewhere in between: being paced at the septal location they probably had less ventricular dyssynchrony compared to apical RVP. It is important to underline that, in our center, all leadless pacemakers are implanted at the mid-septal or high-septal area, resulting in a shorter ventricular activation [30] by potentially recruiting the peripheral Purkinje network located along the right side of the interventricular septum. Furthermore, these patients had a significant amount of time with spontaneous AV conduction due to the presence of an intermittent AV block, thus, resulting in the absence of pacing-induced ventricular dyssynchrony. This combination translates into unchanged echocardiographic parameters compared to the baseline. Our observation confirms the recent report by Vijayaraman et al. [31] on a broader population, thanks to the pathophysiologic interpretation of patients’ outcomes as enabled by the septal placement of LL pacemakers and the extent of RV stimulation.

### 4.2. Usefulness of the Leadless Pacemaker in Clinical Practice

Our observations suggest that the advantage of implanting a leadless pacemaker is greater in patients at high risk of short/medium-term complications following transvenous implantation, especially when infection risk is concerned, as in patients on dialysis or with advanced kidney failure. Unfortunately, the mortality of the sickest patients was impacted by non-cardiovascular events, resulting in a total mortality rate of 50% at three years. As previously noted, leadless VDD technology seems suitable for a niche of patients who rely on pacing less than 20% of the time, or for frail patients, beyond the few lacking superior vein access to the heart. Another advantage of LL is the limited risk of interaction with the tricuspid valve structure, which minimizes the risk of significant regurgitation [32]. The technological evolution of these systems is, however, continuous and will soon shift the focus on designing more advanced devices, such as dual-chamber and VDD leadless pacemakers for conduction system pacing.

### 4.3. Study Limitations

This study is a prospective non-randomized single-center study with a small study population. To mitigate cohort heterogeneity and confounding factors, the selected patients were propensity-score matched based on their baseline characteristics. However, the remaining differences that have guided physicians’ decision-making, namely dialysis, may have potentially influenced the outcome but were not avoidable. Nonetheless, randomized clinical trials are highly unlikely to occur in the future due to the complexity of assessing significant endpoints in an aged population, which is highly vulnerable to non-cardiovascular events [18]. For this reason, data from observational registries will become increasingly important for octogenarians in the future.

## 5. Conclusions

Conduction system pacing offers superior results compared to other pacing modalities in terms of ventricular function owing to a physiological ventricular activation and maintenance of AV synchrony. However, in frail patients or in those without superior venous access, leadless pacing does not seem to cause a significant deterioration in cardiovascular function despite suboptimal AV synchrony, and is proposed as the device of choice in patients at high risk of complications.

## Figures and Tables

**Table 1:** Baseline characteristics of the population.

	Total N = 88	LL N = 22	HBP N = 33	RVP N = 33	*p*-Value
Implant age, years	74.2 ± 10.1	72.2 ± 13.6	75.9 ± 10.6	73.8 ± 6.1	n.s.
Female sex	27 (30.7)	7 (31.8)	12 (36.4)	8 (24.2)	n.s.
Hypertension	69 (78.4)	16 (72.7)	27 (81.8)	26 (78.8)	n.s.
Diabetes	25 (28.4)	8 (36.4)	10 (30.3)	7 (21.2)	n.s.
CKD	27 (30.7)	12 (54.5)	12 (36.4)	3 (9.1)	<0.01
Dialysis	11 (12.5)	9 (40)	2 (6)	0 (0)	<0.01
Coronary artery disease	22 (25.0)	7 (31.8)	8 (24.2)	7 (21.2)	n.s.
Treated valvular heart disease	22 (25)	10 (45)	7 (21)	5 (15)	0.03
NYHA class > 1	51 (58.0)	15 (68.2)	17 (51.5)	19 (57.6)	n.s.
History of paroxysmal/persistent AF	26 (30.0)	11 (45.5)	9 (27.3)	6 (18.2)	0.04

AF = atrial fibrillation, CKD = chronic kidney disease, HPB = His bundle pacing, LL = leadless pacemaker, n.s.= non-significant, RVP = right ventricular pacing.

**Table 2:** Cox proportional hazard models for all-cause mortality.

	Univariate Analysis			Multivariate Analysis		
	HR	95% CI	*p*-Value	HR	95% CI	*p*-Value
Dialysis	9.04	3.47–23.56	<0.001	5.44	1.41–21.10	0.01
Treated valvular heart disease	2.05	0.94–4.49	n.s.			
History of paroxysmal/persistent af	2.21	1.02–4.78	0.04	1.30	0.55–3.08	n.s.
Type of pacemaker						
LL						
HBP	0.32	0.12–0.88	0.03	0.78	0.22–2.84	n.s.
RVP	0.17	0.05–0.53	0.002	0.46	0.10–2.07	n.s.

AF = atrial fibrillation, CKD = chronic kidney disease, HPB = His bundle pacing, LL = leadless pacemaker, n.s.= non-significant, RVP = right ventricular pacing.

**Table 3:** Echocardiographic characteristics at the baseline and one-year follow-up.

	Total (n°88)	LL (n°22)			HBP (n°33)			RVP (n°33)		
	Baseline	Baseline	FUP	*p*	Baseline	FUP	*p*	Baseline	FUP	*p*
LVEF (%)	57.5 ± 10.73	54.0 ± 12.2	53.3 ± 8.3	n.s.	57.2 ±10.1	62.6 ± 7.0	n.s.	60.2 ± 9.93	53.1 ± 12.5	0.001
End diastolic volume, (mL)	109 ± 40.9	118 ± 51.0	120.3 ± 33.0	n.s.	110 ± 35.0	99.1 ± 24.2	0.009	101 ± 38.5	122.3 ± 47.3	<0.001
End systolic volume (mL)	48.3 ± 30.5	58.0 ± 42.8	62.7 ± 19.7	n.s.	49.4 ± 25.1	43.0 ± 14.4	0.014	40.8 ± 23.9	60.6 ± 37.7	<0.001
Left atrial AP diameter (mm)	43.4 ± 7.88	45.5 ± 10.2	49.3 ± 11.3	n.s.	43.7 ± 6.28	42.4 ± 9.2	n.s.	41.4 ± 5.95	42.8 ± 5.7	n.s.
MR > mild (%)	15 (17.0)	6 (27.3)	6 (27.3)	n.s.	5 (15.2)	2 (6.1)	n.s.	4 (12.1)	9 (27.3)	0.008
PAPs (mmHg)	32.6 ± 9.95	34.0 ± 10.3	34.0 ± 12.2	n.s.	32.5 ± 11.1	32.5 ± 12.1	n.s.	31.1 ± 8.31	31.1 ± 10.0	n.s.

AP = antero-posterior, FUP = follow-up, HPB = His bundle pacing, LL = leadless pacemaker, LVEF = left ventricular ejection fraction, MR = mitral regurgitation, n.s.= non-significant, PAPs = systolic pulmonary artery pressure, RVP = right ventricular pacing.

**Table 4:** Peri-procedural and long-term complications.

	Total N = 88	LL N = 22	HIS N = 33	RVP N = 33	*p*-Value
Periprocedural complication (%)					
Pneumothorax	2 (2.3)	0 (0.0)	1 (3.0)	1 (3.0)	n.s.
Pericardial effusion	0 (0.0)	0 (0.0)	0 (0.0)	0 (0.0)	n.s.
Vascular access/pocket complication	1 (1.1)	1 (4.5)	0 (0.0)	0 (0.0)	n.s.
Late-onset complications (%)					
Lead dislodgement	1 (1.1)	0 (0.0)	0 (0.0)	1 (3.0)	n.s.
Device infections	0 (0.0)	0 (0.0)	0 (0.0)	0 (0.0)	n.s.
Threshold increase > 1.75 V @ 0.40 ms	2 (2.3)	0 (0.0)	1 (3.0)	1 (3.0)	n.s.

HBP = His bundle pacing, LL = leadless pacemaker, n.s.= non-significant, RVP = right ventricular pacing.

**Table 5:** Extent of ventricular stimulation over all pacemaker interrogations.

Patients	Total N = 88	LL N = 22	HBP N = 33	RVP N = 33	*p*-Value
% of Ventricular Pacing delivered	82 ± 32	52 ± 22	91 ± 7	92 ± 5	<0.001

HBP = His bundle pacing, LL = leadless pacemaker, RVP = right ventricular pacing.

## Data Availability

The data presented in this study are available on request from the corresponding author due to privacy restrictions.
